# Development of Phase and Seasonally Dependent Land-Use Regression Models to Predict Atmospheric PAH Levels

**DOI:** 10.3390/toxics11040316

**Published:** 2023-03-28

**Authors:** Ayibota Tuerxunbieke, Xiangyu Xu, Wen Pei, Ling Qi, Ning Qin, Xiaoli Duan

**Affiliations:** School of Energy and Environmental Engineering, University of Science and Technology Beijing, Beijing 100083, China

**Keywords:** polycyclic aromatic hydrocarbons, land use regression, seasonal, phase dependent

## Abstract

Polycyclic aromatic hydrocarbons (PAHs) are an important class of pollutants in China. The land use regression (LUR) model has been used to predict the selected PAH concentrations and screen the key influencing factors. However, most previous studies have focused on particle-associated PAHs, and research on gaseous PAHs was limited. This study measured representative PAHs in both gaseous phases and particle-associated during the windy, non-heating and heating seasons from 25 sampling sites in different areas of Taiyuan City. We established separate prediction models of 15 PAHs. Acenaphthene (Ace), Fluorene (Flo), and benzo [g,h,i] perylene (BghiP) were selected to analyze the relationship between PAH concentration and influencing factors. The stability and accuracy of the LUR models were quantitatively evaluated using leave-one-out cross-validation. We found that Ace and Flo models show good performance in the gaseous phase (Ace: *adj.* R^2^ = 0.14–0.82; Flo: *adj.* R^2^ = 0.21–0.85), and the model performance of BghiP is better in the particle phase (*adj.* R^2^ = 0.20–0.42). Additionally, better model performance was observed in the heating season (*adj* R^2^ = 0.68–0.83) than in the non-heating (*adj* R^2^ = 0.23–0.76) and windy seasons (*adj* R^2^ = 0.37–0.59). Those gaseous PAHs were highly affected by traffic emissions, elevation, and latitude, whereas BghiP was affected by point sources. This study reveals the strong seasonal and phase dependence of PAH concentrations. Building separate LUR models in different phases and seasons improves the prediction accuracy of PAHs.

## 1. Introduction

Polycyclic aromatic hydrocarbons (PAHs) comprise two or more benzene rings bonded in linear, cluster, or angular arrangements [1]. They are of public concern as typical persistent toxicants in the air because of their persistence, toxicity, bioaccumulation, and their significant carcinogenic, teratogenic, and mutagenic effects [2]. In addition, PAHs are strongly associated with several adverse health effects [3], and long-term exposure to low doses of PAHs is highly likely to cause skin, lung, bladder, and gastrointestinal cancers [4]. PAHs mainly originate from the incomplete combustion of fossil and biomass fuels [5]. PAHs are a major pollutant in China but have not been included in the list of pollutants in routine monitoring due to high monitoring costs and instrumentation requirements. The lack of monitoring data, especially on gaseous-phase PAHs, makes it difficult to assess the health risks associated with PAH exposure.

Land-use regression (LUR) models are typically established with the assumption of a linear relationship between predicted variables and predictors such as traffic, land use, topography, and meteorology [6]. This model reveals the statistical correlation between pollutants and prediction information and analyzes the factors affecting their changes. LUR-based empirical models have been used in many studies owing to their complete consideration, relatively convenient data acquisition, high spatial resolution, and wide applicability [7,8]. Considerable studies have been conducted to establish LUR models for the routine monitoring of pollutants, including nitrogen dioxide, PM_2.5_, and ozone [6,9,10,11,12] Modeling studies of PAHs have gradually emerged in recent years. A European study modeled particle-bound PAHs based on 10 one-year samples collected from different areas [13]. The models for each study area were developed based on the annual average concentrations. Four variables explained approximately 67 and 71% of the variances in benzo[a]pyrene (BaP) and chrysene, respectively. White et al. [14] developed nationwide PAH models in the Czech Republic using data from 29 sites, wherein the PAH model was an annual model of the total concentrations of 14 PAHs.

Additionally, several city-scale LUR models have also been studied [13,15,16]. Most previous research has focused on particle-bound PAHs, and less attention has been paid to gaseous-phase PAHs [13,17,18]. Second, only a few studies have reported seasonally dependent models. Finally, comparisons among different PAHs have seldom been conducted. As typical semi-volatile organic compounds, PAHs are present in both particulate and gaseous phases in the atmosphere. The high concentration of gaseous PAHs in the atmosphere can also pose certain health risks to the human body. Moreover, the gas-particle partitioning behavior was seriously affected by seasonal factors, such as temperature, humidity, precipitation, and particle concentration, as well as by PAHs’ physical and chemical properties, such as lipophilicity. Therefore, multiple influencing factors, especially the seasonal and phase dependence of PAH concentration, should be considered in a LUR model of PAHs.

In this study, the concentration of 15 priority-controlled PAHs in 25 sampling sites was collected and measured in Taiyuan, a typical northern industrial city in China. Based on land-use regression (LUR) analysis, separate prediction models for each phase and season were established. Three PAHs, including acenaphthene (Ace), Fluorene (Flo), and benzo [g,h,i] perylene (BghiP), were selected to study the influencing factors. The stability and accuracy of the LUR models were quantitatively evaluated using leave-one-out cross-validation. This research aims to validate the reliability and goodness of the season and phase dependence LUR model on PAHs. Our study will provide information for the future application of LUR models on the concentration of PAHs.

## 2. Materials and Methods

### 2.1. Study Area

**Figure 1:**
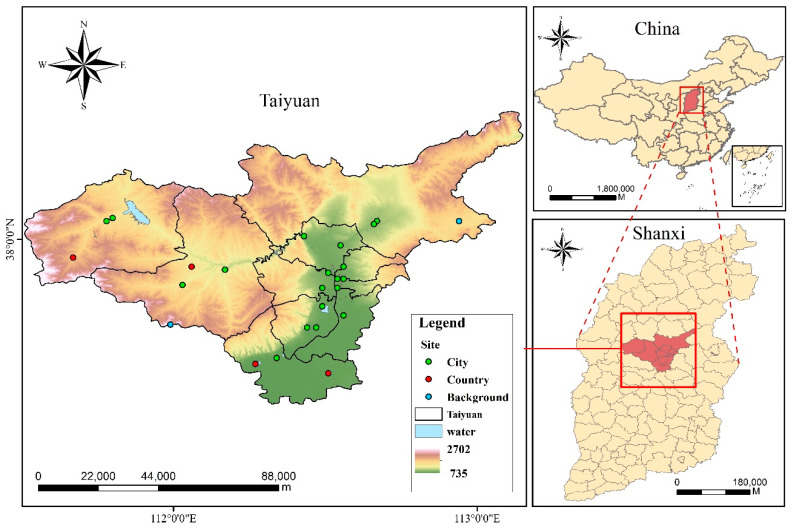
Location of the study area.

Taiyuan (37° 27′–38°25′ N, 111°30′–113°09′ E) is located in the interior of China and is surrounded by mountains on three sides, with valley plains in the central and southern parts. Taiyuan has a typical warm temperate continental monsoon climate, controlled by the Siberian cold air mass in winter and influenced by the humid and hot air mass in the southeast ocean during the summer. Spring is susceptible to the alternating effects of these two air masses, which produce dusty weather. It significantly contributes to the transport of atmospheric pollutants. Owing to the presence of resources and historical reasons, the industrial structure of Taiyuan is dominated by traditional heavy pollution industries, such as metallurgy, coal coke, machinery, chemical industry, and electric power, and its energy structure is dominated by coal. A previous study found that Taiyuan emitted approximately 332.1 tons of PAHs in 2010 [19]. The daily average concentration of BaP in the atmosphere was 23.88 ng/m^3^, which is more than 9 times higher than the limit of the Chinese standard (daily limit is 2.5 ng/m^3^, GB 3095-2012, phased-in 2012–2016) [20]. Additionally, the sources of PAH pollution have changed from coal soot pollution to motor vehicle emissions and coal combustion. The study area and sampling locations are shown in Figure 1.

### 2.2. Sampling and Analytical Methods

Pollutant concentration data were collected during the windy season (70 days) from April to June 2009, the non-heating season (153 days) from July to November 2009, and the heating season (120 days) from December 2009 to March 2010. PAH samples bound to atmospheric PM_10_ and in the gaseous phase were collected from three sampling sites in each region using passive air samplers from 2009 to 2010 (Figure 1). A total of 25 sites included 19 urban sites, 4 rural sites and 2 background sites. Two samples were collected at the same time at each site. The blank samples were analyzed along with the other samples. PAH samples in the gas and particulate phase were extracted, purified, and measured using an Agilent 6890 N gas chromatograph coupled with an Agilent 5975 mass spectrometer with an electron impact ion source. The mass spectrometer works in scan mode and selects ion detection mode. The pretreatment method and quantification procedures have been previously described [21,22,23]. The details of the sampling and instrument analysis were provided in SI. Fifteen priority-controlled PAHs were identified, including acenaphthene (Ace), acenaphthylene (Acy), fluorene (Flo), phenanthrene (Phe), anthracene (Ant), fluoranthene (Fla), pyrene (Pyr), benz(a)anthracene (BaA), chrysene (Chr), benzo(b)fluoranthene (BbF), benzo(k)fluoranthene (BkF), benzo(a)pyrene (BaP), dibenz(a, h) anthracene (DahA), indeno(1,2,3-cd)pyrene (IcdP), and benzo(ghi) perylene (BghiP).

### 2.3. Variables for LUR

All the predictive variables are presented in Table 1. Data land cover types are divided into 11 types: plough, forest, grassland, shrub, wetland, water, tundra, artificial surface, bare land, glaciers, and permanent snow cover. There were six types of land use: plough, forest, grassland, shrub, wetland, water, urban and rural and unutilized. The road length variable was divided into motorways, primary roads, and non-motor vehicles. As for the data on contaminated sites, we divided it into the number of contaminated sites within 5000 m and the distance to the nearest factory. Geographical factors were divided into elevation, longitude, and latitude. The rainfall was divided into daytime rainfall and nighttime rainfall. Other meteorological variables include pressure, relative humidity, temperature, and wind speed. Among them, forest, grassland, *shrub*, wetland, and tundra are widely believed to be negatively correlated with pollutant concentration, while artificial surface, urban and rural, motorways, primary roads, the number of contaminated sites within 5000 m and the distance to the nearest factory considered to be positively correlated with pollutants. The road is a sign of a traffic emission source, and the road length will directly affect the traffic emission in the region. Taiyuan is an industrial city, and the number of factories and distance also contributed.

Road data was derived from OpenStreetMap (2009–2010). According to the data description, road variables were divided into highways, main roads, and non-motorized lanes. Circular buffer zones were established to collect variables; the monitoring points were set at the center of the circle, and circular buffer zones of 500–5000 m were established. The total lengths of highways, main roads, and all roads in the buffer zones were calculated using ESRI ArcGIS 10.6 (ESRI, Inc., Redlands, CA, USA). The predictive variables representing traffic conditions were obtained. Meteorological data were derived from the daily dataset of surface climatic data from the China Meteorological Science Data Sharing Service Network (http://data.cma.cn/, accessed on 12 March 2021). From April 2009 to June 2010, the daily observation data of 36 basic meteorological stations in the study area were obtained, including 5 items such as atmospheric pressure, precipitation, temperature, wind speed and relative humidity. Averaged meteorological data in three sampling seasons were obtained by calculating the arithmetic mean value of daily meteorological data. Statistical data were imported into ArcGIS10.6, the grid data of meteorological elements in the study area were obtained by the Kriging interpolation method, and grid values at air quality monitoring points were extracted as meteorological factors. Land-use data were obtained from the global land-use dataset of Tsinghua University in 2017 (http://data.ess.tsinghua.edu.cn/, accessed on 24 December 2020). Demographic data from the World Pop project (https://www.worldpop.org/, accessed on 24 December 2020) data sets, including the data since 2000 on the World’s population. With a spatial resolution of 100 m, the value of each grid represented one hectare within the scope of the population. The time period selected in this study was 2010, and the population density data of the study area were obtained by using the clipping of the vector boundary of the Taiyuan administrative region. Finally, according to different buffer sizes, the population number of the corresponding buffer was obtained. Data on industrial pollution sources were obtained from the official website of the Shanxi Provincial Department of Ecology and Environment (https://sthjt.shanxi.gov.cn/, accessed on 4 April 2021). Geographic data, including elevation, latitude, and longitude, were provided by the Geospatial Data Cloud site of the Computer Network Information Center, Chinese Academy of Sciences. (http://www.gscloud.cn, accessed on 12 March 2021).

### 2.4. Land Use Regression Model Development

Based on ArcGIS 10.6, the average seasonal concentration of PAHs and 30 predictive variables of 25 monitoring sites in the Taiyuan administrative region were selected to establish the LUR model for three periods: the windy, non-heating, and heating seasons. To reduce the possibility of collinearity between variables belonging to the same category and ensure the interpretability of model parameters, we followed the method used in the European Study of Cohorts for Air Pollution Effects (ESCAPE) study. First, all the prediction variables were provided separately, and the variable with the highest R^2^ and the slope in the specified direction was selected. Second, if the adjustment R^2^ of the model was increased by at least 1% and the influence direction was the same as the prior decision, variables were individually added to the model based on the highest adjustment R^2^. R version 4.0.4 was used for the data statistics, selection and validation of predictors, and estimation of pollutant concentrations. We examined the following aspects during the fitting process: (1) significance tests for individual variables (*p* < 0.05); (2) collinearity test for the variance inflation factor (VIF < 5); and (3) Cook’s distance (D value < 1) and model residual space autocorrelation (Moran’s I).

### 2.5. Model Validation and Mapping

Owing to the limited number of monitoring sites, leave-one-out cross-validation was used to evaluate the model’s accuracy. The 25 sites were randomly divided into two parts: 24 experimental sets and one validation set. The model estimates of the remaining samples were calculated and compared to the actual PAH concentrations at the sample site. This process was repeated 25 times to obtain the simulation accuracy, and root mean square error (RMSE) of the LUR model for the study area. After developing the final LUR model, the pollutant concentrations at non-monitoring sites were estimated using a regression equation, which can better simulate the spatial variation of pollutants mechanistically than spatial interpolation. To predict the spatial distribution of PAH concentrations in Taiyuan, a regular grid of 500 × 500 m (1815 in total) was established in this study. The values of each predictor variable in the regression equation were calculated for each grid point and substituted into the regression equation to obtain the concentration value of PAHs at each grid point. The spatial distribution of the mass concentration of PAHs in Taiyuan was obtained by pan-Kriging interpolation based on the data of each centroid.

## 3. Results and Discussion

### 3.1. Descriptive Statistics

**Figure 2:**
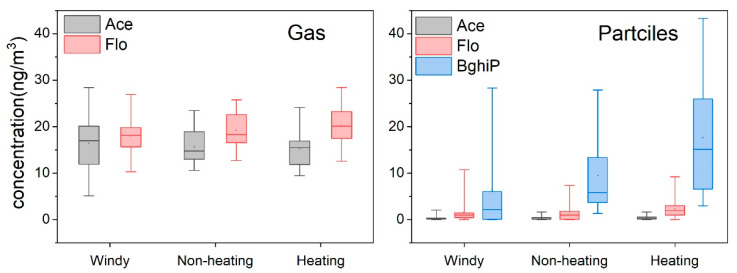
Statistical comparison of PAHs concentration (ng/m^3^) between gas and particle phases in the atmosphere. The squares represent means, and the solid lines represent median values. Boxes enclose the interquartile range, and whiskers show the full range.

The monitoring data from 25 sampling sites showed that 15 PAHs were detected in gaseous and particulate samples. 81LUR models of 15 PAHs in three sampling seasons were established (Table S1). The PAHs with the least (Ace) and greatest molecular weight (BghiP) were selected to analyze the influencing variables due to their difference in physiochemical properties. Flo was also selected because it contains the most variables and has the best goodness of fit, which means higher model accuracy and reliability. The gaseous Ace and Flo detection rates were 100% in all three seasons. In contrast, the detection rate for the particulate phase was approximately 91%. Gaseous BghiP was not detected in any of these samples. The concentrations of Ace, Flo, and BghiP obtained from the mobile monitoring survey are shown in Figure 2. In the gaseous phase, the mean Ace concentration was 16.42 ng/m^3^ (median: 16.96 ng/m^3^) in the windy season, which was slightly higher than those in the heating (15.24 ng/m^3^; median: 15.49 ng/m^3^) and non-heating seasons (15.65 ng/m^3^; median: 14.73 ng/m^3^). The mean Flo concentration showed different seasonal trends, with mean concentrations of 20.22 ng/m^3^ (median: 20.12 ng/m^3^), 19.18 ng/m^3^ (median: 18.31 ng/m^3^), and 18.03 ng/m^3^ (median: 118.13 ng/m^3^) during the heating, non-heating, and windy seasons, respectively. In the particulate phase, the Ace concentration was relatively low. The Flo and BghiP concentrations showed similar seasonal trends. Higher concentrations were observed during the heating season, and lower concentrations were observed in the non-heating and windy seasons. PAHs may have a higher concentration in the heating season than in the non-heating season due to the combustion of coal and biomass. Kruskal-Wallis test was used to detect the difference between light PAHs (Ace and Flo) in heating and non-heating seasons. As a result, no significant difference was observed with both *p* > 0.05. the result is consistent with data reported in Guangzhou [24]. A potential reason may be gaseous PAH concentrations increase with temperature, suggesting evaporation of these light PAHs from the contaminated ground surface under higher ambient temperatures [25]. Other studies have shown that low molecular weight PAHs may be originated from non-seasonal sources, such as vehicle exhaust, and industrial sources may also cause higher Ace and Flo concentrations in the non-heating season than in heating seasons [26].

The upper and lower limits of the box plots represent the divergence of the sites, and the inter-site concentration varied over time. Overall, the differences in gas-phase concentrations were smaller than in particle-phase concentrations, indicating a larger spatial variation.

### 3.2. Models and Validation

In the 15 models of the three target PAHs, the adjusted R^2^ varied from 0.14 to 0.85. The models and their parameters are listed in Table 2. All six Ace models of the gaseous phase in the heating and non-heating seasons had better fitting results. Six variables were entered into the gas phase model in the non-heating season, including road variables, latitude, and elevation. The high-speed variables and artificial surfaces showed a positive correlation, and latitude, elevation, non-motorized road length, and water reservoirs showed a negative correlation with the Ace concentration. The main variables that entered the gas model during the heating season were grass, altitude, and the length of the non-motorized driveway. Notably, in the corrected model in the windy season, the only variable that entered the model was the artificial surface within the 2000 m range (lc2000_80). This variable showed a positive correlation with the Ace concentration. Compared to that of the gaseous phase, the simulation of the particulate phase was poor. The variable that entered the particulate phase model during the non-heating season was the distance to the pollution source. In the windy season, point sources had a positive influence on Ace emissions, whereas, in the heating season, latitude had a negative influence on Ace emissions.

The corrected R^2^ of gaseous Flo varied from 0.70 to 0.88. The R^2^ of gaseous Flo was considerably better than the particulate phase models. This is similar to the simulation of Ace; however, Flo has a higher molecular weight than Ace. In the windy season, three variables entered the gaseous phase model of Flo, including the length of the non-motorized road within 2000 m, latitude, and forest within 5000 m. All three variables were negatively correlated with the Flo concentration, and only the number of pollution point sources within 5000 m entered the particulate phase mode. In the non-heating season, six variables were entered into the gas-phase model, including road variables, altitude, and grass. The number of point sources was positively correlated with the Flo concentration.

Meanwhile, the distance to the pollution source had a positive effect on the particulate Flo concentration. During the heating season, the latitude, elevation, and length of non-motorized vehicles within 500 m and artificial surfaces within 1500 m were entered into the gas-phase model. However, only the latitude was entered into the particle-phase model and showed a negative correlation with the Flo concentration.

Owing to the low detection rate of gaseous BghiP, only particle-phase models were developed. Residential land in the 2500 m range appeared to be the most important variable influencing the concentration of BghiP. This variable existed in all three models of particulate BghiP and was the only factor influencing BghiP concentrations in the heating and non-heating seasons. BghiP has long been considered a typical marker for diesel vehicles [27,28]. With increasing residential land use, traffic changes associated with anthropogenic activities are significantly correlated with residential land use. They could, therefore, also explain the use of only one variable in the model. Moreover, non-motorized roadways in the 500 m range were another factor influencing the model in the windy season but showed a negative effect on BghiP emissions.

The average fit accuracy R^2^ value of the relevant LUR model studies published worldwide until 2018 was approximately 0.6 [29]. The significance levels of the regression equation F test variance analysis were less than 0.01, indicating that the equation was highly significant. The linear relationship between the predictive variables that entered the equation and pollutant concentration was very close. The adjusted R^2^ of the gas-phase Ace model ranged from 0.76 to 0.87. Ace was widely available in the gas phase form; therefore, the fit of the particle phase model was low, and the explanatory power of the model was relatively weak. The low R^2^ value of the windy season model can be attributed to the wind and sand caused by air mass interactions. The adjusted R^2^ value of the Flo gas-phase model was 0.66–0.85, and that of the particle-phase model was 0.21–0.39, which was higher than that of Ace. The adjusted R^2^ of the BghiP model was in the range of 0.20–0.42. The poor explanatory power of the particle-phase model may be due to the complex factors affecting PAHs in the particle phase, especially in winter.

The RMSE of the final results obtained by the leave-one-out cross-validation method is also presented in Table 2. There is no specific criterion to define the RMSE value range, and the smaller the value, the higher the model’s accuracy. In similar tests of previous studies, the value range was stipulated to be within 15 for better accuracy [30,31]. The verification showed that most of the resulting models had good accuracy.

### 3.3. Model Performance in Different Phases and Seasons

**Figure 3:**
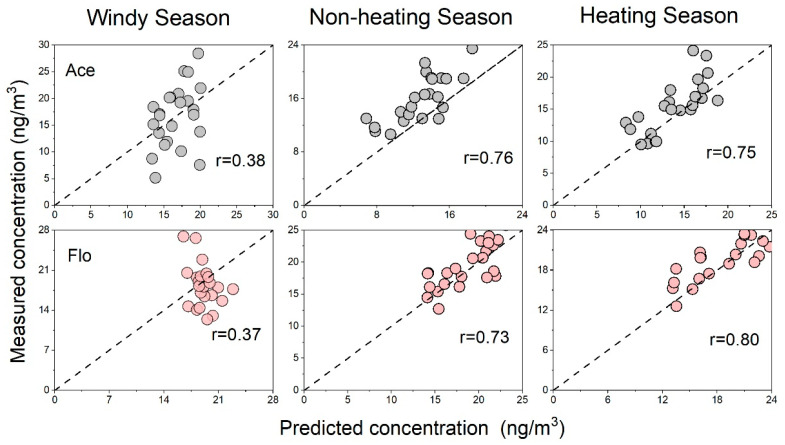
Results of leave-one-out cross-validation for gaseous PAHs LUR models: predicting concentrations (*x*-axis) against measured concentrations (*y*-axis) for LUR models.

**Figure 4:**
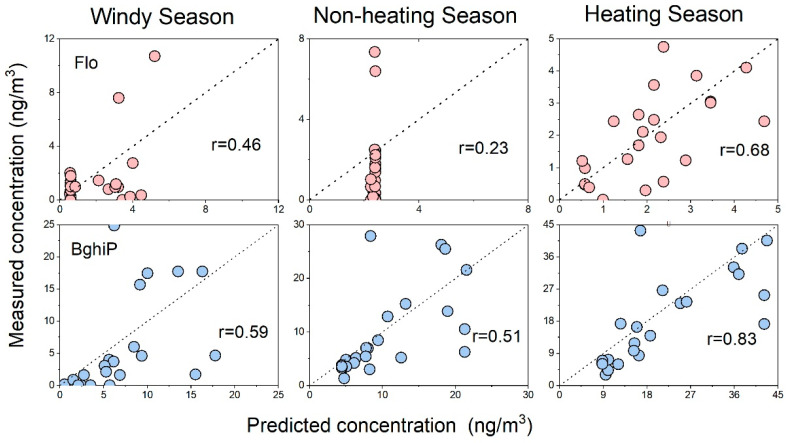
Results of leave-one-out cross-validation for particulate PAHs LUR models: predicting concentrations (*x*-axis) against measured concentrations (*y*-axis) for LUR models.

Figure 3 and Figure 4 represent the model performance in different phases. As a low-molecular-weight PAH, Ace mainly existed in the atmosphere in the gas phase, and the particle phase model was poorly fitted owing to the limited detection rate. Therefore, the models of particle-bounded Ace were not discussed. In contrast, Bghip was mainly associated with particles. Obvious differences in performance were observed in the different phases of Flo.

Generally, the LUR models of Flo showed better performance under gaseous conditions, wherein the correlation coefficients (r) varied from 0.37 to 0.80. In contrast, relatively lower accuracy was observed in the particulate phase of Flo, wherein the r values varied from 0.23 to 0.68. Notably, in the equations, more variables were selected for the gaseous phase models than for the particulate phase models. In the gas-phase Flo model, the non-motor lane entered each model, followed by the latitude and elevation, which entered the three models. We proved that traffic variables, latitude, and elevation greatly influenced the Flo concentrations. The forest variable only existed in the windy season model, which might be attributed to seasonality [32]. For the particle phase models, Flo concentration in the windy season was highly related to the polluted sites. However, in the non-heating season, the only predicted variable for the heating season was latitude. This result was probably due to the unique terrain of Taiyuan. The terrain of Taiyuan is generally low in the south and high in the north, and the latitude has the same interpretation significance as altitude to a certain extent. It is blocked by the surrounding mountains, and the pollutant concentration is mainly affected by vertical diffusion. A comparison of the observed and simulated concentrations revealed that the point concentration in Qingxu was the highest, which has also been demonstrated in previous studies [19].

According to the seasonal variation, the windy season showed a large deviation, which may have led to an obvious change in the air mass. Therefore, the selected variables could not satisfactorily identify the pollution source. The other two seasons showed a good fit, wherein the heating season showed the best fit. The fit of the particle phase was significantly better during the heating season than during the other two seasons. The highest Flo concentration in the heating season gas-phase model was 26.76 ng/m^3^. Qingxu is located in the southernmost part of the Taiyuan administrative region, with flat terrain and the lowest altitude among the administrative regions, thus resulting in a trend of pollutant transport from north to south. It caused serious pollution controlled by local circulation, explaining the variables selected in the model. The three pollutant contributing sources in the heating and non-heating seasons were point sources, artificial surfaces, and major lane lengths, which confirmed that Flo originated from anthropogenic heating and traffic emissions.

The Ace model showed significantly different seasonal variations. It is most likely due to the low precipitation in Shanxi in spring and the windy weather caused by the combined effect of cold air and Mongolian cyclones, resulting in higher Ace concentrations in the air during the windy season than during the heating season. An artificial surface within 2000 m was selected to provide a positive contribution to the windy season gas-phase model. However, in the full model of the windy season, the relative humidity and the water area within 3500 m were introduced, and the sandstorm was negatively correlated with the relative humidity [33]. Relative humidity could reduce PAH concentrations by accumulating atmospheric particulate matter [34]. The octanol-water partition coefficient (Kow) is an important indicator determining the gas-particle partition. Generally, the Kow of PAHs increases with the number of rings. This implies that high-molecular-weight PAHs are more inclined to be adsorbed on the particles; thus, their proportion in the gas phase would gradually decrease. Comparing the gas-phase models for the heating and non-heating seasons revealed two important positive contributions in the gas-phase model for the non-heating period: the length of high-speed roads within 3000 m and the artificial surface within 1500 m. The length of the highway represents the traffic variable [13]. In contrast, the area of the artificial surface was used to represent the degree of urbanization, including sidewalks, roads, buildings, factories, and airports. These structures are made of impenetrable materials, such as roofs, asphalt, brick, and stone [35]. The number of key monitoring enterprises in the gas-phase model for the heating period was introduced into the model, providing a positive contribution. However, the temperature decreased in winter, and some heating enterprises produced more exhaust gases than in other seasons, increasing pollutant concentrations. It can be seen that good simulation results were achieved for gaseous Ace and Flo in the heating season and particulate BghiP in three seasons. The performance of the LUR model and the results of validation confirm the reliability and accuracy of the phase and season dependence strategy.

### 3.4. Comparison among PAHs

In addition to the prediction of phase and seasonal variation, the prediction of LUR models can also be affected by the physiochemical properties of different PAHs. Similarities can be found between Ace and Flo by comparison between gaseous-phase LUR models. The particle and phase models were associated with variables such as contaminated sites, highways, elevation, and water body variables in the meteorological model of the non-heating period. Among them, highway and major lanes displayed positive contributions, whereas non-motorized lanes, elevation, grass, and water bodies contributed to some extent to the degradation of PAH concentrations. All n gas-phase models in the heating season were related to non-motorized lanes and elevation. The elevation factor showed a strong negative correlation with the gas-phase PAH concentrations, which was absent in the meteorological model during the windy season, indicating that the elevation of the monitoring station had a great influence as a point source of pollution, especially in summer when it had the greatest influence on the regional PAH concentration. Highways and main roads showed positive correlations, representing tailpipe emissions from motor vehicle operation and dust from traffic emissions, which was similar to a study conducted in the United States [13]. These variables correspond to the industrial uses of Ace and Flo. Ace is a component of crude oil and a by-product of coking production, and Flo is mainly derived from automobile exhaust emissions, straw burning, and industrial production [36].

The comparison among the LUR models of Ace, Flo, and BghiP revealed that the goodness of the particulate phase models increased, whereas that of the gaseous models decreased with an increase in the molecular weight of PAHs. To a large extent, this can be attributed to the gas-particle partition of PAHs. High-molecular-weight PAHs with high octanol-water partition coefficients prefer to be bound in particles. In this case, predicting the gaseous phase content through the model is not valuable. In contrast, it is the same for gaseous prediction models of PAHs with low Kow values. In addition, contaminated sites were included in the particle-phase model of Flo for all seasons, whereas all three seasonal models of BghiP included sites generating BghiP by anthropogenic activities, and only the BghiP model for the windy season included non-motorized roads within 500 m.

To some extent, this explains the emission sources of these two substances. Flo has a closer relationship with burning and emissions from contaminated sites, whereas BghiP was related to mobile emissions from anthropogenic activities. With increasing residential sites, the changes in traffic from anthropogenic activities were significantly correlated with residential sites and could be explained as only one variable in the model. From the location distribution of the sampling points, we observed that the denser the sampling points, such as the urbanized central area, the more accurate the predicted value.

### 3.5. Mapping of PAHs in Taiyuan and Key Influencing Factors

**Figure 5:**
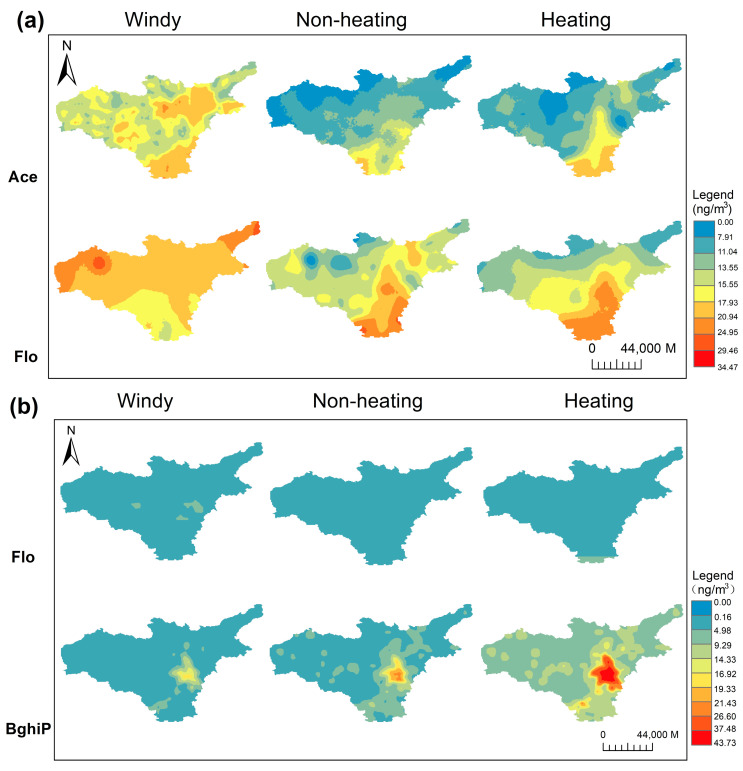
LUR exposure surfaces in three seasons (from left to right: windy season, non-heating season, heating season). Where (**a**) represents PAHs in the gas phase and (**b**) represents PAHs in the particulate phase.

The distribution of PAHs is shown in Figure 5. The PAH concentration showed significant spatial heterogeneity. The high-concentration enrichment areas of Ace, Flo, and BghiP were concentrated in the urban area and the surrounding suburbs, especially in the south, where the concentration was even higher than that in the urban area. The reasons for this phenomenon may be related to the topography, meteorological factors, regional transport of pollutants, and the different vegetation cover and land-use types in the substrate. The operation of motor vehicles, construction of bare ground, and dust could increase the PAH concentrations [37].

LUR models were used to evaluate the data for different seasons. The elevation parameter is a strong determinant of predicted pollutant concentrations [18]. The results indicated the necessity of calculating the elevation of the predicted points in most of the gas-phase models because in Taiyuan, the terrain had a distinct characteristic variation, and the pollutant concentrations were higher at low elevations and low latitudes. In addition, PAHs have short atmospheric lifetimes due to the deposition and photochemical degradation of particulate matter [38]. Therefore, they are strongly influenced by the emission altitude, exacerbated by topographic factors in the study area [39]. The R^2^ value indicates that the LUR prediction was better in the heating season than in the non-heating season, probably because of the relative stability of emission sources during the heating season.

Meteorological conditions were also considered in previous studies. Both temperature inversion and wind speed magnitude are significant factors influencing the spatial concentration distribution of air pollutants, but these processes show short-term variations. Our study was modeled with seasonally averaged concentrations; therefore, no air-phase factors entered our model, which is perhaps one of the reasons for the poor fit of our model.

The results of the LUR model in the study area showed that the predicted values were closer to the measured values in places with a dense number of monitoring points, such as the urban center of Taiyuan, whereas fewer points were observed in mountainous and rural areas. Therefore, traffic variables were selected as important influencing factors in our model. In addition, due to the lack of traffic flow data, our model used road length as a variable, consistent with previous studies [40,41].

### 3.6. Limitations

PAH data in this research were collected during 2009–2010. PAH concentration may be different from the previous PAH level in Taiyuan. However, we believe our innovative study will provide the necessary information for further research of LUR application on PAHs. The aim of this paper is not limited to regression model development for predicting atmospheric PAH concentrations ten years ago but also focuses on the validation of the availability and goodness of the LUR model on PAHs. Modeling the centent of PAHs can be difficult due to the influence of the environment on PAH properties. Therefore, it is necessary to make more detailed and specific models. Excluding the influence of physical and chemical factors, LUR for individual PAH is more accurate in results and more reasonable in the method. This research is the first attempt at the application of season and phase-dependent strategies for PAHs. Based on the data obtained, We will carry out a new data collection plan, and our future research will focus on the PAH concentration prediction Taiyuan.

## 4. Conclusions

Taiyuan, an important industrial city in China, has been widely affected by PAH pollution. In this study, we established seasonal- and phase-dependent LUR models. Gaseous PAHs Ace and Flo were mainly affected by traffic, altitude, and latitude. BghiP, a typical member of PM-associated PAHs, was mainly affected by human activities. Considering seasonal differences, the goodness of fit was better in the heating season than in the non-heating and windy seasons. Our study revealed the seasonal and phase dependence of the studied PAH concentrations. According to the LUR models of three PAHs developed in this study, it is necessary to continue establishing other separate LUR models in different phases and seasons to improve the prediction efficiency and obtain optimal results.

## Figures and Tables

**Table 1:** Potential variables with units, defined buffer sizes and priority-defined directions of the effect.

Type	Potential Variables	Group Code	Buffer Size	Unit	Coefficient Sign Setting
Land cover (the total area in the buffer)	Plough	lc_10	500–5000	m^2^	/
	Forest	lc_20	500–5000	m^2^	-
	Grassland	lc_30	500–5000	m^2^	-
	Shrub	lc_40	500–5000	m^2^	-
	Wetland	lc_50	500–5000	m^2^	-
	Water	lc_60	500–5000	m^2^	-
	Tundra	lc_70	500–5000	m^2^	-
	Artificial surface	lc_80	500–5000	m^2^	/
	Bare land	lc_90	500–5000	m^2^	/
	Glaciers and permanent snow cover	lc_100	500–5000	m^2^	/
Land use (the total area in the buffer)	Plough	lu_1	500–5000	m^2^	/
	Forest	lu_2	500–5000	m^2^	-
	Grassland	lu_3	500–5000	m^2^	-
	Water	lu_4	500–5000	m^2^	-
	Urban and rural	lu_5	500–5000	m^2^	-
	Unutilized	lu_6	500–5000	m^2^	/
Water	Water	w	500–5000	m^2^	-
Road length (total length in buffer)	Motorway	r_51	500–5000	m	+
	Primary roads	r_52	500–5000	m	+
	Non-motor vehicle	r_53	500–5000	m	
Point feature	Number of factories within 5000 m	point	N/A	N/A	+
	Distance to the nearest factory	dis	N/A	m	+
Geographic information	Elevation	dem	N/A	m	/
	Longitude	long	N/A	N/A	/
	Latitude	lat	N/A	N/A	/
Precipitation	Daytime average	rain_8	N/A	mm	/
	Nighttime	rain_20	N/A	mm	/
Pressure	average	pre	N/A	hPa	/
Relative humidity	Daytime average	hum	N/A	%	/
Temperature	Daytime average	tem	N/A	N/A	/
Wind speed	Daytime average	wind	N/A	N/A	/

Note: (1) “/” means the sign is uncertain.

**Table 2:** LUR models for Ace, Flo, and BghiP in Taiyuan.

PAH	Season	Phase	LUR Model	R^2^	*adj.* R^2^	RMSE
Ace	Windy season	Gaseous phase	6.71 × 10^−7^ lc2000_80 + 13.11	0.180	0.140	5.124
		Particle phase	2.49 point−0.22	0.374	0.347	1.822
	Non-heating season	Gaseous phase	5.63 × 10^−5^ r3000_51-10.32 lat − 0.01 dem − 10^−4^ r3000_53 + 8.63 × 10^−7^ lc1500_80 − 3.60 × 10^−6^ lc3000_60 + 420.82	0.863	0.818	1.287
		Particle phase	0.518−1.73 × 10^−5^dis	0.346	0.219	0.332
	Heating season	Gaseous phase	−1.44 × 10^−6^ lu2000_3 − 8.22 × 10^−3^ dem − 1.4 × 10^−4^ r2000_53 + 1.84 dis	0.800	0.760	1.803
		Particle phase	−1.29 lat + 49.4	0.262	0.230	15.215
Flo	Windy season	Gaseous phase	−3.36 × 10^−7^ lc5000_20 − 1.55 lat − 1.71 × 10^−4^ r2000_53 + 609.65	0.7	0.657	2.101
		Particle phase	2.51 point−0.61	0.363	0.336	1.883
	Nonheating season	Gaseous phase	−8.82 × 10^−3^ dem − 4.12 × 10^−7^ lu3500_3 − 1.52 × 10^−4^ r2500_53 + 1.92 point + 8.5 × 10^−5^ r1500_52 − 2.23 × 10^−6^ lc3000_60 + 31.81	0.884	0.846	1.210
		Particle phase	2.71–8.09 × 10^−5^ dis	0.245	0.212	1.580
	Heating season	Gaseous phase	−6.8 × 10^−3^ dem − 13.66 lat + 6.86 × 10^−6^ lc1500_80 − 8.06 × 10^−4^ r500_53 + 5.42 × 10^2^	0.835	0.802	1.638
		Particle phase	−8.21 × lat + 3.13 × 10^2^	0.412	0.387	1.468
BghiP	Windy season	Gaseous phase				
		Particle phase	9.02 × 10^−7^ lu2500_5 − 1.57 × 10^−3^ r500_53 + 2.12	0.471	0.423	6.091
	Nonheating season	Gaseous phase				
		Particle phase	4.42 lu2500_5 + 4.42	0.295	0.265	9.256
	Heating season	Gaseous phase				
		Particle phase	1.72 × 10^−6^ lu2500_5 + 8.85	0.235	0.202	21.385

## Data Availability

Not applicable.

## Supplementary Materials

The following are available online at https://www.mdpi.com/article/10.3390/toxics11040316/s1, Text S1 Sampling and analytical methods, Table S1: LUR models for PAHs [22,42,43].
