# Supplementary material for: Development of Phase and Seasonally Dependent Land-Use Regression Models to Predict Atmospheric PAH Levels
Source: Toxics. 2023 Mar 28;11(4):316. doi: 10.3390/toxics11040316 (PMC10145409; doi:10.3390/toxics11040316)
Supplement: Supplementary file 1 [file toxics-11-00316-s001.zip › toxics-2239897-supplementary.pdf]

toxics

Article

Development of Phase and Seasonally Dependent Land-Use Regression Models to Predict Atmospheric PAH Levels

Ayibota Tuerxunbieke, Xiangyu Xu, Wen Pei, Ling Qi, Ning Qin * and Xiaoli Duan *

School of Energy and Environmental Engineering, University of Science and Technology Beijing, Beijing 100083, China
* Correspondence: qinning@ustb.edu.cn (N.Q.); jasmine@ustb.edu.cn (X.D.)

List of tables

Text S1 Sampling and analytical methods

Table S1: LUR models for PAHs.

Text S1. Sampling and analytical methods

PAHs in gas and particle phases were collected by a PUF disk and a glass fiber filter (GFF) passive air sampler in 25 sampling sites from 2009 to 2010. Five sites with different air pollution levels were selected for sampler calibration. At each study calibration site, two identical passive air samplers and one active air sampler (PM10A-300, Guangzhou Institute of Geochemistry, Chinese Academy of Sciences, Guangzhou, China) were used to collect PAHs in gaseous and particulate phases. The detailed calibration method and uptake rate of the passive sampler have been described previously [42, 22]. Prior to sampling, PUF discs were extracted for 8 hours in a Soxhlet solvent 1:1 between n-hexane and acetone, cleaned, and GFFs discs were baked for 4 h in a furnace at 450 °C [43].

PUF discs were extracted by Soxhle extraction at 52 °C with a 1:1 mixture of 150 mL hexane and acetone for 8 h. GFFs were extracted by microwave with 25 mL of a 1:1 mixture of hexane and acetone (MARS2Xpress, CEM, USA), heated to 100 °C at 10 °C /min, and then

maintained for 10 min. Surrogate standards of 2-fluoro-1, 1′-biphenyl and p-terphenyl-d14 (J&K Chemical, USA, 2.0 mg•mL^{-1}) were added to the samples to indicate the recovery before extraction After concentration with a vacuum rotary evaporator (R-201, Shanghai, China) at 37℃, the elution solution was, conversed to hexane solution, and then added with internal standards (Nap-d8、Ace-d10、Ant-d10、Chr-d12 and Perylene-d12, J&K Chemical Ltd., USA). The total extract was transferred to an alumina silica gel column for purification. During purification, the mixture eluted from the column was first concentrated to near dryness. The residue was then transferred, diluted with hexane, and made accurate to 1.0 mL by nitrogen discharge (Eyela MG-1000) at room temperature (25 °C). Samples were sealed in vials and stored at −4 °C prior to analysis. Air sample extracts were quantified using a gas chromatography-mass spectrometry detector (Agilent 6890GC/5973MSD). A 30 m × 0.25 mm i.d × 0.25 μm film thick HP-5MS capillary column (Agilent Technology) was used. The GC temperature was set at 5 ℃/min from an initial 60 °C to 280 °C with a final holding time of 20 min. Helium was used as the carrier gas. At a flow rate of 1.0 mL/min, 1.0 μl of extract was injected into the injection port in a crack-free mode at 280 °C . The pressure of the first column was 30 kPa. The mass spectrometer was operated in scan mode with electron impact ionization of 70 eV, electron doubling voltage of 1288 V, and ion source of 230 °C . Concentrations of 15 pahs were determined in all samples. They are acenaphthene (Ace), acenaphthylene (Acy), fluorene (Flo), phenanthrene (Phe), anthracene (Ant), fluoranthene (Fla), pyrene (Pyr), benz(a)anthracene (BaA), chrysene (Chr), benzo(b)fluoranthene (BbF), benzo(k)fluoranthene (BkF), benzo(a)pyrene (BaP), dibenz(a, h) anthracene (DahA), indeno(1,2,3-cd)py rene (IcdP), and benzo(ghi) perylene (BghiP).

All solvents used were purified by distillation before use (Beijing Chemical Reagent, Beijing, China), Alumina and silica gel (80–200 mesh; Dikma, China) were heated in a muffle furnace (DLII-9, Beijing, China) at 650 °C for 10 h and stored in a sealed desiccator. It was reactivated at 130 °C for 4 h prior to use. All glassware was cleaned using an ultrasonic cleaner (KQ-500B, Kunshan, China) and heated at 400°C for 6 hours. The results of all air samples were blank corrected in the field. Recovery rates for individual PAHs range from 78% to 103%, with a mean of 89% for PUF disks, and 80% to 101% for GFFs disks with a mean of 88%. The data analyzed in this paper did not correct the recovery rate. The detection limit was 0.172 ~ 1.23 ng/mL .

Table S1: LUR models for PAHs

PAH	Season	Phase	LUR model	R^2	*adj*.R^2	RMSE [a]
Acy	Windy season	Gaseous phase	$6.81-5.84\times10^{-6}$lc3500_50[b]	0.30	0.27	1.967
		Particle phase	$9.90\times10^{-2}+0.44$point [c]	0.23	0.21	0.495
	Non-heating season	Gaseous phase	$7.60+7.90\times10^{-5}$r4500_51 [d]$+4.06\times10^{-4}$r1000_53 [e] -8.39×10^{-5}ppl_1000 [f] -5.97×10^{-6}lc3000_50	0.80	0.75	2.332
		Particle phase	$3.22\times10^{-1}-7.01\times10^{-8}$lu2000_3[g]$+1.52\times10^{-1}$point	0.48	0.43	0.204
	Heating season	Gaseous phase	$10.06-6.95\times10^{-7}$lu2000_3$+1.11$point -1.01×10^{-7}lc500_30 -2.87×10^{-5}r3000_53-5.80×10^{-5}dis [h]	0.87	0.83	1.002
		Particle phase	$3.96\times10^{-1}-8.40\times10^{-8}$lu2000_3	0.34	0.31	0.241
Phe	Windy season	Gaseous phase	$24.88-3.78\times10^{-3}$dem [i]	0.35	0.32	2.724
		Particle phase	$27.25+1.50\times10^{-3}$ppl-3.66×10^{-4}r3500_53$+15.35$point-1.23×10^{-7}lu3500_3-2.30×10^{-5}lc4500_50	0.70	0.66	2.985
	Non-heating season	Gaseous phase	$13.33+5.29\times10^{-5}$r500_51	0.64	0.62	4.832
		Particle phase	$11.71-1.07\times10^{-6}$lu3000_3	0.35	0.32	7.091
	Heating season	Gaseous phase	$3.61-7.01$dem-3.30×10^{-6}lu1000_3$+2.40$point-3.40long [j] -2.62×10^{-6}r500_53	0.89	0.85	3.001
		Particle phase	$22.35-1.52\times10^{-6}$lu3000_3	0.41	0.38	8.835
Ant	Windy season	Gaseous phase	$6.34-5.48\times10^{-6}$lc500_20 [k]	0.19	0.16	1.198
		Particle phase	$1.11+1.84$point	0.23	0.19	2.216
	Non-heating season	Gaseous phase	$5.52+4.95\times10^{-6}$r4500_51	0.52	0.50	1.596
		Particle phase	0.39rain_20 [l] $-20.23-1.88\times10^{-7}$lu3000_3	0.52	0.47	0.958
	Heating season	Gaseous phase	$181.62+4.14\times10^{-6}$lc500_80 [m] -2.73×10^{-6}lc1000_30-4.66lat [n] -2.93×10^{-4}r500_53	0.87	0.84	1.513

		Particle phase	219.76-5.75lat	0.39	0.36	1.210
Fla	Windy season	Gaseous phase	$4.69+2.01\times10^{-7}$lc2000_80	0.32	0.29	1.210
		Particle phase	$9.09+2.64\times10^{-4}$ppl	0.28	0.25	2.899
	Non-heating season	Gaseous phase	$3.79+2.38\times10^{-5}$r500_51-2.52×10^{-6}lc3500_50	0.71	0.69	0.989
		Particle phase	$4.10+1.60\times10^{-4}$r4500_51	0.38	0.35	11.940
	Heating season	Gaseous phase	$133.21-3.31\times10^{-3}$dem-8.58×10^{-8}lu3500_3-1.70×10^{-4}r500_53-2.77×10^{-6}lu500_3-1.11long+0.41point	0.89	0.85	0.574
		Particle phase	$8.20+1.96\times10^{-6}$lu2500_5^{o}	0.45	0.42	16.201
Pyr	Windy season	Gaseous phase	$3.90+9.33\times10^{-8}$lc2500_80	0.31	0.28	0.922
		Particle phase	$5.75+1.45\times10^{-4}$ppl	0.24	0.20	13.159
	Non-heating season	Gaseous phase	$3.16+1.91\times10^{-5}$r5000_51-2.14×10^{-6}lc3500_50	0.71	0.69	0.805
		Particle phase	$13.18-2.81\times10^{-6}$lu2000_3	0.34	0.32	7.878
	Heating season	Gaseous phase	$84.77+3.42\times10^{-7}$lc1500_80-1.51×10^{-3}dem-2.11lat-2.47×10^{-5}r2000_53	0.80	0.76	0.586
		Particle phase	$4.47+9.35\times10^{-7}$lu2500_5	0.36	0.33	9.205
BaA	Windy season	Gaseous phase	$2.21+4.90\times10^{-7}$lu2500_5	0.20	0.16	7.231
		Particle phase	8.35-2.81×10-4dis	0.24	0.21	5.924
	Non-heating season	Gaseous phase	$0.34+2.70\times10^{-6}$ppl	0.52	0.50	0.150
		Particle phase	$5.56-1.73\times10^{-4}$dis	0.25	0.21	3.582
	Heating season	Gaseous phase	$0.30+3.30\times10^{-8}$lc1500_80	0.61	0.57	0.074
		Particle phase	$2.20+4.89\times10^{-7}$lu2500_5	0.20	0.16	7.231
Chr	Windy season	Gaseous phase	3.14+9.12×10-7lu2500_5	0.31	0.28	10.091
		Particle phase	$13.89-4.65\times10^{-4}$dis	0.2	0.17	10.989

	Non-heating season	Gaseous phase	0.44+3.86×10^{-6}ppl	0.64	0.63	0.176
		Particle phase	2.05+5.44×10-7lu2500_5	0.30	0.27	6.120
	Heating season	Gaseous phase	0.62+1.83×10^{-8}lc200_80-1.73×10^{-4}dem-1.74×10^{-5}r500_53	0.71	0.67	0.072
		Particle phase	3.14+9.12×10-7lu2500_5	0.31	0.28	10.091
BbF	Windy season	Gaseous phase	0.04+1.01×10^{-8}lu2500_5	0.23	0.20	0.021
		Particle phase	7.71+3.27×10^{-6}lu2500_5-3.62×10^{-4}r3500_53	0.46	0.41	16.478
	Non-heating season	Gaseous phase	0.05+6.31×10^{-7}ppl	0.60	0.57	0.035
		Particle phase	4.93+1.14×10^{-6}lu2500_5	0.33	0.30	12.030
	Heating season	Gaseous phase	0.05+3.78×10^{-9}lc2000_80	0.55	0.53	0.014
		Particle phase	9.21+2.17×10^{-6}lu2500_5	0.30	0.27	24.250
BkF	Windy season	Gaseous phase	0.04+5.44×10^{-9}lu2000_5	0.23	0.20	0.015
		Particle phase	2.94+1.01×10^{-6}lu2500_5-1.26×10^{-4}r3500_53	0.42	0.36	5.185
	Non-heating season	Gaseous phase	0.03+3.54×10^{-7}ppl	0.51	0.48	0.024
		Particle phase	1.29+4.95×10^{-7}lu2500_5	0.43	0.41	4.253
	Heating season	Gaseous phase	0.07-9.92×10^{-10}lu3500_2	0.49	0.46	0.011
		Particle phase	2.86+8.47×10^{-7}lu2500_5	0.31	0.28	9.389
BaP	Windy season	Gaseous phase	0.013+4.15×10^{-7}r4000_51+7.53×10^{-9}lu1000_1	0.51	0.44	0.011
		Particle phase	7.95-6.92×10^{-7}lu300_3	0.21	0.18	6.394
	Non-heating season	Gaseous phase	0.03+3.78×10^{-7}ppl	0.53	0.51	0.025
		Particle phase	2.89+4.72×10^{-7}lu2500_5	0.22	0.19	6.392
	Heating season	Gaseous phase	0.03+1.42×10^{-9}lu2500_5	0.47	0.45	0.011
		Particle phase	3.60+8.81×10^{-7}lu2500_5	0.19	0.15	13.390
DahA	Windy season	Gaseous phase	-			

		Particle phase	5.69×10^{-7}lc2000_80-0.26-5.05×10^{-5}r2000_52	0.45	0.4	1.590
	Non-heating season	Gaseous phase	-			
		Particle phase	3.17-1.01×10^{-4}dis	0.26	0.23	1.990
	Heating season	Gaseous phase	-			
		Particle phase	1.79+2.78×10^{-7}lu2500_5	0.19	0.15	4.170
IcdP	Windy season	Gaseous phase	-			
		Particle phase	7.51×10^{-7}lc2500_80-0.48	0.34	0.31	7.056
	Non-heating season	Gaseous phase	-			
		Particle phase	2.41+6.26×10^{-7}lu2500_5	0.32	0.29	6.779
	Heating season	Gaseous phase	-			
		Particle phase	4.66+1.25×10^{-6}lu2500_5	0.26	0.22	15.648

Note: [a] root mean square error

[b] Wetland

[c] Number of factories within 5000m

[d] Motorway

[e] Non-motor vehicle

[f] Population

[g] Grassland

[h] Distance to nearest factory

[i] Elevation

[j] Longitude

[k] Forest

[l] Nighttime Precipitation

[m] Artificial surface

[n] Latitude

[o] Urban and rural

References:

1. Tao, S.; Cao, J.; Wang, W.; Zhao, J.; Wang, W.; Wang, Z.; Cao, H.; Xing, B. A passive sampler with improved performance for collecting gaseous and particulate phase polycyclic aromatic hydrocarbons in air. Environmental Science and Technology 2009, 43, 4124-4129, doi:10.1021/es900522v.

2. Xia, Z.H.; Duan, X.L.; Tao, S.; Qiu, W.X.; Liu, D.; Wang, Y.L.; Wei, S.Y.; Wang, B.; Jiang, Q.J.; Lu, B.; et al. Pollution level, inhalation exposure and lung cancer risk of ambient atmospheric polycyclic aromatic hydrocarbons (PAHs) in Taiyuan, China. Environmental Pollution 2013, 173, 150-156, doi:10.1016/j.envpol.2012.10.009.

3. MacKay, D.; Shiu, W.Y.; Ma, K.C. Volume III, volatile organic chemicals. In *Illustrated Handbook of Physical Chemical Properties and Environmental Fate of Organic Chemicals*; Lewis Publishers: Boca Raton, FL, USA, 1993; 916p, ISBN 0-87371-973-5.
